# The public’s irrational use of antibiotics for upper respiratory tract infections: a cross-sectional study based on the health belief model

**DOI:** 10.1038/s41598-025-01767-9

**Published:** 2025-05-17

**Authors:** Xi Wang, Chenxi Liu, Shuangjiang Zheng, Xinyi Zhang, Rujiao Lin, Lixia Duan, Dan Wang, Qianning Wang, Weidong Zhong, Xin Ding

**Affiliations:** 1 School of Medicine and Health Management, Tongji Medical School, Huazhong University of Science and Technology, Wuhan, Hubei China; 2 Major Disciplinary Platform Under Double First-Class Initiative for Liberal Arts at Huazhong, University of Science and Technology (Research Center for High-Quality Development of Hospitals), Wuhan, Hubei China; 3 School of Management, Hubei University of Chinese Medicine, Wuhan, Hubei China; 4 Union Hospital Tongji Medical College Huazhong University of Science and Technology, Tongji Medical School, Huazhong University of Science and Technology, Wuhan, Hubei China; 5 Department of Medical Affairs, The First Affiliated Hospital of Chongqing Medical University, Yu Zhong District, Chongqing, China

**Keywords:** Antibiotics, Irrational use, Health belief model, Public, Structural equation modeling, Risk factors, Disease prevention, Drug regulation, Health policy, Public health

## Abstract

To understand the reasons for the public’s irrational use of antibiotics based on the health belief model (HBM). A questionnaire survey was conducted based on cluster random sampling in Chongqing, China. The public’s antibiotic use behaviors, knowledge, perceived threat of diseases [both short-term upper respiratory tract infections (URTIs) and long-term antibiotic resistance (AR)], perceived value of antibiotic use (benefits and harm), self-efficacy, antibiotic availability and social influences were measured. Structural equation modeling (SEM) was applied to test the fitness of the survey data with the theoretical framework based on the HBM. A total of 815 respondents were enrolled. The irrational use of antibiotics was prevalent among the public (mean: 2.95, SD = 2.12). The public had limited knowledge about antibiotic use (average 29.17% correct answers to 8 questions), a high perceived threat of AR (mean = 2.46, SD = 0.64) and a moderate perceived threat of URTIs (mean = 2.13, SD = 1.04). They also perceived high benefits (mean = 2.57, SD = 0.68) and moderate harm (mean = 2.16, SD = 0.83) from antibiotic use. In addition, respondents had easy access to antibiotics (mean = 2.38, SD = 0.80), perceived a high prevalence of use of antibiotics by relatives (mean = 2.40, SD = 0.65) and had a moderate level of self-efficacy in using antibiotics (mean = 1.97, SD = 0.75). The SEM results showed that higher levels of the perceived threat of URTIs, perceived benefits of antibiotic use, self-efficacy, antibiotic availability and social influence were associated with more irrational antibiotic use behavior (p < 0.005). Moreover, higher knowledge indirectly led to irrational use of antibiotics by promoting self-efficacy (p < 0.001) and the perceived threat of URTIs (p < 0.005). To curb the irrational use of antibiotics, improving knowledge alone is insufficient. A ​​systematic approach addressing multiple dimensions of health beliefs​​ is critical. This includes: (1) ​​targeted public education campaigns​​ emphasizing the limited efficacy of antibiotics for viral infections and reframing perceptions of antibiotic “benefits”; (2) ​​regulatory measures to restrict non-prescription antibiotic sales​​ in pharmacies; (3) ​​clinical guidelines and training​​ to reduce unnecessary antibiotic prescriptions by healthcare providers; and (4) ​​community-level interventions​​ leveraging social norms to discourage inappropriate antibiotic use. Policymakers should prioritize interventions that address both individual perceptions (e.g., fear of untreated infections) and systemic drivers (e.g., antibiotic accessibility).

## Introduction

Antibiotic resistance (AR) has become one of the greatest threats to global public health and economic development, endangering our ability to treat common infectious diseases and leading to increased mortality and treatment costs. It has been reported that approximately 700,000 people die from AR worldwide every year. Without effective countermeasures, this figure is estimated to reach 10 million by 2050, leading to USD $100 trillion in economic losses worldwide^1^. It is likely that the misuse and overuse of antibiotics contribute to the problem of AR^2–4^. However, the inappropriate use of antibiotics is still prevalent worldwide, especially in developing countries^5^.

Despite studies that have focused on inappropriate prescriptions of antibiotics by health care providers, recent systematic reviews highlight that the general population also plays a key role in the misuse and overuse of antibiotics worldwide^6–9^, including nonprescription antibiotic purchasing^10^, antibiotic self-medication, the preventive use of antibiotics, and the demand for antibiotic prescriptions from health care providers. Although antibiotics are commonly prescribed-required worldwide^11,12^, a global systematic review showed that more than 50% of people self-medicate with antibiotics in most regions of the world^13^.This situation is especially severe in low-income and middle-income countries^14,15^. Therefore, reducing the public’s irrational use of antibiotics has been included as a core measure in global, regional and national action plans to address AR.

However, the reasons why people use antibiotics inappropriately are still unclear. A systematic review shows that inappropriate outpatient and community antibiotic use is influenced by non-biomedical factors at the individual, community, health system and societal levels^16^. While the knowledge-attitude-practice (KAP) model has been widely applied to study antibiotic misuse, its utility depends critically on contextual conditions. In settings where behavioral change relies predominantly on individual agency (e.g., hand hygiene with accessible facilities), KAP model demonstrates predictive validity^17^. However, this linear knowledge-to-behavior assumption becomes inadequate when structural barriers (e.g., antibiotic accessibility) or conflicting social norms override rational decision-making—a phenomenon explained by the "risk compensation effect": informed individuals may overestimate their self-efficacy in managing infections, accelerating inappropriate use^18,19^.

A cross-sectional study indicates that recognizing the severity of antimicrobial resistance (AMR) positively impacts the appropriate use of antibiotics^20^. While the KAP model or other frameworks do not account for this factor, the Health Belief Model (HBM) adequately addresses it. Recent studies increasingly adopt the health belief model (HBM), which extends KAP model by integrating mediators between knowledge and behavior^21^. The HBM accounts for competing perceptions (e.g., weighing immediate symptom relief against long-term antibiotic resistance risks), socio-environmental cues (e.g., social normalization of misuse), and structural triggers (e.g., non-prescription access). It has been used in a recent systematic review and showed potential to guide further understanding of the reasons for the public’s irrational use of antibiotics. Several studies have referred to the HBM to reanalyze secondary data in which proxy indicators of key constructs in the HBM were generated, such as knowing that upper respiratory tract infections (URTIs) are self-limiting as a measure of the perceived severity of diseases. However, the validity and reliability of these indicators are unclear, resulting in a lack of clarity regarding the reasons for the public’s irrational use of antibiotics^22–25^.

As one of the world’s largest consumers, China has long been criticized for its excessive and irrational use of antibiotics. The general population contributes significantly to the overuse and misuse of antibiotics in China^26^. A study conducted among university students showed that the inappropriate use of antibiotics due to the demand side (the public and patients) has surpassed the supply side (health care providers) to become the main contributor to the irrational use of antibiotics in China^16,23^. Several recent systematic reviews also confirmed that roughly half of the general population in China purchases antibiotics without a prescription and more than one-third of the general population requires their doctors to prescribe antibiotics^10,27^. This situation is more severe in the western region due to intersecting structural vulnerabilities. First, fragmented healthcare access—western China has 28% fewer primary clinics per capita than eastern regions, forcing residents to rely on unregulated pharmacies for urgent care. Second, lax enforcement of prescription policies enables 63% of rural western pharmacies to dispense antibiotics without prescriptions, compared to less than 30% in Zhejiang (the eastern region)^10^.

This study aimed to understand the reasons for the public’s irrational use of antibiotics for URTIs based on the health belief model.

## Participants and methods

### Settings

This study was conducted in Chongqing Province, China, a region representative of midlevel socioeconomic development in western China^28^. Chongqing reports high antibiotic prescription rates (37.72% of outpatients in 2020), exceeding WHO recommendations (30%)^29^. Findings from a systematic review and meta-analysis suggest that people from western China are more likely to exhibit behaviors that lead to unnecessary antibiotics. It has been reported that 65% of the general population from western China asked doctors to prescribe antibiotics, 70% purchased antibiotics without a prescription, 47% did not adhere to antibiotic prescriptions, and 47% used antibiotics prophylactically to prevent infections^27^. These behaviors may be influenced by socioeconomic disparities between urban and rural areas, which can affect access to healthcare and information. In rural regions, limited access to qualified healthcare providers and lower health literacy often lead to an increased reliance on informal antibiotic procurement and non-adherence to prescribed treatments.

### Theoretical framework

The health belief model (HBM) was used as the theoretical basis in the current study. According to the HBM, people’s knowledge, perceived threat of disease, perceived value of behaviors and social influence (cues to action) are potential determinants of health behaviors^16,30^. In terms of perceived threat of disease, research shows that the public may consider the threat of short-term URTIs and the threat of long-term AR in their decision-making regarding antibiotic use^19^. Regarding the perceived value of behaviors, people may evaluate both the potential benefits and the potential harms of antibiotic use when deciding whether such behavior is valuable^30,31^. In addition, studies have highlighted the effect of antibiotic availability and personal self-efficacy with antibiotics on antibiotic use behaviors^30,32^ and have suggested that self-efficacy may play a mediating effect between knowledge and behaviors^24,33^.

**Fig. 1:**
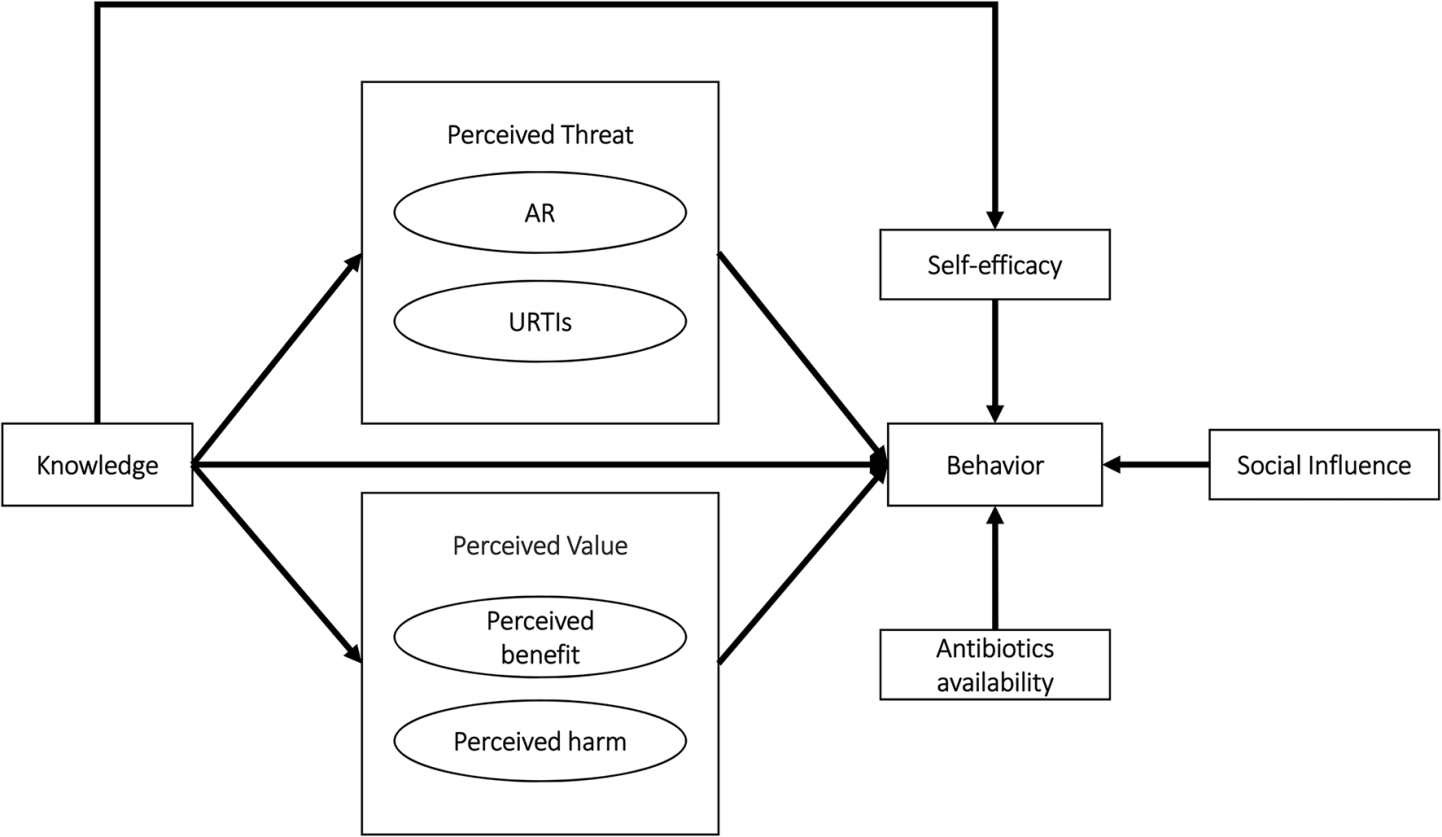
Theoretical framework based on the HBM.

Therefore, we hypothesized that the public’s antibiotic use is directly influenced by individual antibiotic use knowledge, the perceived threat of AR and URTIs, the perceived value of antibiotic use (including potential benefits and harms), antibiotic use self-efficacy, antibiotic availability and social influence. In addition, people’s knowledge can indirectly influence antibiotic use behaviors via changes in antibiotic use self-efficacy, the perceived threat of diseases and the perceived value of behaviors. The specific behavioral model is shown in Fig. 1.

### Sampling and participants

The sample size was calculated based on the following formula:

$$n=\frac{{{Z}_{\alpha /2}}^{2}\times p\times (1-p)}{{\delta }^{2}}\times [1+(M-1)\times ICC]$$

Previous studies have shown that the prevalence of the public’s irrational antibiotic use behaviors ranges from 36.5% to 81.8%^27^. Considering α (significance level) = 0.05, δ (tolerance) = 0.05, M (number of participants in each cluster) = 35, and intraclass correlation coefficient (ICC) = 0.02, the sample size was required to be larger than 646. We estimated that the invalid response rate would range from 10 to 12%, so the final sample size was calculated as 735, with 21 clusters^34^.

Two-step cluster random sampling was adopted in the current study with the community (urban area) and county (rural area) as clusters. In the first stage, Yuzhong District, Tongnan District, and Chengkou District were selected randomly, respectively representing high, middle, and low socioeconomic levels in Chongqing. In the second stage, seven to nine primary care institutions (urban community health centres and rural township health centres) were randomly selected using a computer-generated random number list from all primary care institutions in each area. This resulted in a sample of 23 institutions. All eligible patients visiting the selected institutions on the survey date were consecutively enrolled to minimize selection bias. If the daily enrollment fell below the target sample size per cluster (minimum 35 participants), the survey period was extended to subsequent days until the quota was met.

The inclusion criteria for the participants were as follows: (1) aged over 18 years old; (2) experienced symptoms of URTIs (sore throat, cough, runny nose, fever, etc.) in the past six months; (3) were able to read and comprehend the survey and were able to complete the survey themselves or with the assistance of investigators; and (4) were not inpatients due to URTIs or diagnosed with lower respiratory tract infections.

This study adhered to the Declaration of Helsinki, and the study obtained ethics approval from the Research Ethics Committee of Tongji Medical College, Huazhong University of Science and Technology (No [2020]-S099). Informed consent was obtained from all subjects involved in the study. This study followed the Strengthening the Reporting of Observational Studies in Epidemiology (STROBE) statement.

### Survey instruments

Based on existing survey instruments, interview materials, and findings from the literature review, a 51-item questionnaire (for details of the survey instrument, see the supplementary files) was developed that included three parts: (1) irrational antibiotic use behaviors for URTIs; (2) determinants of irrational antibiotic use behaviors (derived from the HBM; see Fig. 1); and (3) demographic characteristics.

Respondents’ irrational antibiotic use behaviors were measured with nine items that were derived from existing instruments or drafted based on an existing study^19^. Potential irrational antibiotic use behaviors were addressed, including antibiotic demand, prophylactic antibiotic use, antibiotic self-medication, nonprescription purchasing of antibiotics, nonadherence to antibiotic prescriptions and storing and sharing antibiotics. Respondents were asked whether they had performed these irrational antibiotic use behaviors for URTIs in the past year, and binary responses were used (yes and no).

Knowledge of antibiotics was measured using eight true or false questions that asked respondents to judge whether statements about antibiotics were correct. The knowledge items were derived from existing studies and covered indications for antibiotic use, causes of AR, and misconceptions about antibiotics and AR^18,35^. To discourage guessing, an option of “unknown” was added.

The perceived threat of URTIs and AR was measured by three and five items, respectively. Respondents were asked to answer to what extent they perceived URTIs and AR as a threat^36^. For example, “Antibiotic resistance will threaten the health of myself and my family” or “I often worry that cold may make me badly ill”. For the perceived value of antibiotics, both the benefits and harm of antibiotics were measured with four and three items, respectively. People’s misconceptions that antibiotics could shorten the course of disease and reduce complications were considered the perceived benefits of antibiotics, and the adverse effects of antibiotics were considered the perceived harm of antibiotics^37^. In terms of antibiotic use self-efficacy, five items measured respondents’ confidence in their ability to rationally use antibiotics^38–40^, such as, “I think I have enough knowledge to rationally use antibiotics”. Two items examined the social influences on antibiotic use behaviors and measured the extent to which physicians, pharmacists, families, and social norms influence people’s antibiotic use^22^, such as, “Friends or family recommended that I use antibiotics to treat a cold”. Regarding the availability of antibiotics, three items assessed how easily antibiotics could be obtained from pharmacies, family members and friends^41^. A five-point Likert scale was adopted for the responses to items measuring the above constructs. Finally, the respondents’ demographic characteristics were collected, including age, gender, education, income, occupation, medical background, medical insurance, chronic disease status and self-assessment of health status.

The development of the questionnaire followed the guidelines of the HBM survey and the validation procedure of survey instruments^42,43^. A double translation process was applied to ensure consistency between the original English items and the translated Chinese version. A pilot study was adopted to confirm the face validity and content validity of the questionnaire. Seventeen participants were asked to complete the questionnaire and provide feedback, resulting in the revision, addition, and removal of several items. The validity and reliability of the questionnaire were confirmed in the final survey (n = 735) through confirmatory factor analysis (CFA) and Cronbach’s alpha. The CFA results demonstrated a good fit of the data into the hypothetical model^44,45^: root mean square error of approximation (RMSEA) = 0.072 (< 0.08); Tucker–Lewis index (TLI) = 0.937 (> 0.90); and comparative fit index (CFI) = 0.945 (> 0.90). High internal consistency was evident as indicated by Cronbach’s alpha for the constructs measured in the questionnaire (α: 0.65–0.89). The Cronbach’s alpha for irrational use of antibiotics was 0.70, and the Cronbach’s alpha for knowledge of antibiotics was 0.74.

### Data collection

Postgraduate students in social science and medicine were recruited as investigators. After 1 day of intensive training on the questionnaire, survey procedures and skills, a simulated survey test was conducted, and the investigators who passed the test were eligible to participate in the formal survey. A total of ten investigators were enrolled. For each sampled community or county, paired trained investigators recruited eligible participants. Each survey took 20 min to complete on average, and written informed consent was obtained from the respondents. For quality control, the completeness and consistency of the returned questionnaires were checked on the spot by the investigators. Questionnaires that contained missing items or all responses that were the same were excluded. A token gift (roughly $1.65) was given to the participants. The formal survey was conducted from July to August 2022. A total of 955 questionnaires were distributed and 908 were returned. Finally, 815 responses (response rate: 85.34%) passed the quality control assessment and were included for further analyses.

### Data analysis

Respondents’ irrational antibiotic use behaviors were assessed based on their responses to the nine behavior items. For each item, a response of yes represented irrational use of antibiotics (coded as 1) and vice versa (coded as 0). The overall score of each respondent and the percentage of respondents who showed irrational use of antibiotics were calculated.

Respondents’ knowledge of antibiotics was assessed by the total correct answers to the eight antibiotic knowledge items. For each item, a correct response was coded as 1 and an incorrect/unknown response was coded as 0. The percentage of respondents who gave correct answers to each question and the total number of correct answers per respondent were calculated.

For other characteristics, responses were given on 5-point Likert scales (perceived threat of URTIs and AR, perceived benefits and harm of antibiotics, antibiotic use self-efficacy, social influences and antibiotic availability). Responses were coded as 0 to 4, with higher scores representing higher levels of the measured constructs (for example, a higher perceived threat of URTIs and higher perceived benefits of antibiotics). For each construct, the average score of all items measuring the corresponding construct was calculated (range 0–4).

The differences between the respondents in their potential determinants were examined using paired t tests. Structural equation modeling (SEM) was applied to model the mechanism of the public’s irrational antibiotic use behaviors based on the HBM (see Fig. 1). Means and variance adjusted weighted least squares (WLSMV) estimation were adopted, which were designed for ordinal data (e.g., five-point Likert scales)^46^. The fitness of the data into the SEM was assessed using several recommended criteria^44,45^: RMSEA < 0.08; TLI > 0.90; and CFI > 0.90. The modification index was used to help modify the hypothesized HBM model. Furthermore, the demographic characteristics of the respondents were included to adjust for potential confounding to confirm the robustness of the SEM. Subgroup analysis was also conducted, and an SEM was established for each irrational antibiotic use behavior.

The statistical analyses were performed using STATA (version 12.0) (StataCorp., College Station, TX, USA) and Mplus (version 6.0) (Muthén & Muthén, Los Angeles, CA, USA). A p value < 0.05 was considered statistically significant.

## Results

### Characteristics of respondents

The 815 respondents had a mean age of 46.24 years (standard deviation, SD = 16.76), and most (60.86%) were female. Most of the respondents (70.80%) had no more than a high school or technical secondary school education. On average, the annual household income of the respondents was between 30,000 and 80,000 yuan (4175.95- 11,135.86 USD; based on exchange rate: 1 USD = 7.1846 CNY). Almost half of the respondents or their family members had chronic diseases (Table 1).

**Table 1:** Characteristics of respondents.

Characteristics	Mean ± SD*/N (%)
Age (years)	46.24 ± 16.76
< 40	307 (37.67)
40 ~ 65	371 (45.52)
> 65	137 (16.81)
Gender	
Male	319 (39.14)
Female	496 (60.86)
Level of education	
Primary school or below	275 (33.74)
Junior high school	188 (23.07)
High school or technical school	114 (13.99)
College or above	238 (29.20)
Medical background	
With medical background	144 (17.67)
Without medical background	671 (82.33)
Annual household income (Chinese CNY ¥)	
< 20,000	224 (27.48)
20,000 ~	159 (19.51)
40,000 ~	119 (14.60)
≥ 60,000	313 (38.40)
Insurance	
Basic medical insurance for urban employees	228 (27.98)
Basic medical insurance for urban and rural residents	226 (27.73)
New rural cooperative medical insurance	342 (41.96)
Else	19 (2.33)
Chronic disease	
Yes	404 (49.57)
No/Not aware	411 (50.43)
Subjective health status	
Excellent	20 (2.45)
Good	61 (7.48)
Average	199 (24.42)
Fair	249 (30.55)
Poor	286 (35.09)

*SD Standard deviation.

### Irrational antibiotic use behaviors of respondents

In terms of behaviors, the irrational use of antibiotics was prevalent among the public (Table 2), with respondents commonly showing an average of 3 kinds of irrational use behaviors for antibiotics (mean: 2.95, SD = 2.12). Among them, early cessation of antibiotic use when feeling better (76.56%), keeping antibiotics at home (45.03%), purchasing nonprescription antibiotics from pharmacies (38.90%), and self-medication with antibiotics before care-seeking (35.09%) were the most common behaviors. In contrast, less than one-fifth of the respondents asked doctors for antibiotics (15.09%) or increased their dose of antibiotics themselves (13.25%) due to poor effects.

**Table 2:** Irrational antibiotic use behaviors.

Irrational antibiotic use behaviors	No/Not aware	Yes
1. Use antibiotics to prevent a cold from getting worse	620 (76.07%)	195 (23.93%)
2. Take antibiotics before seeing a doctor	529 (64.91%)	286 (35.09%)
3. Purchase antibiotics from pharmacies without a prescription	498 (61.10%)	317 (38.90%)
4. Demand that your doctor prescribe antibiotics	692 (84.91%)	123 (15.09%)
5. Increase dose of antibiotics by yourself due to poor effect	707 (86.75%)	108 (13.25%)
6. Decrease dose of antibiotics by yourself due to concerns about side effects	650 (79.75%)	165 (20.25%)
7. Stop taking antibiotics when you feel better	191 (23.44%)	624 (76.56%)
8. Keep antibiotics at home	448 (54.97%)	367 (45.03%)
9. Share (receive/give) antibiotics with family, friends, and colleagues	593 (72.76%)	222 (27.24%)

### Characteristics of the measured constructs based on the HBM

The respondents showed poor knowledge of antibiotics (Table 3), with only two knowledge questions answered correctly on average (mean: 2.33, SD: 1.7, total scores: 8). The respondents commonly misunderstood that the body develops resistance to antibiotics (91.41%), that antibiotics are effective in treating viral colds (87.48%) and that antibiotics are anti-inflammatory drugs (84.54%).

**Table 3:** Knowledge of respondents about antibiotic use.

Knowledge questions	True	False	No idea
1. Antibiotics can effectively treat most colds	424 (52.02%)	152 (18.56%)	239 (29.33%)
2. Antibiotics are anti-inflammatory drugs	532 (65.28%)	126 (15.46%)	157 (19.26%)
3. Antibiotics are effective in treating viral colds	427 (52.39%)	102 (12.52%)	286 (35.09%)
4. Antibiotics are effective in treating bacterial colds	404 (49.57%)	69 (8.47%)	342 (41.96%)
5. The human body becomes resistant to antibiotics	465 (57.06%)	70 (8.59%)	280 (34.36%)
6. Bacteria can become resistant to antibiotics	341 (41.84%)	80 (9.82%)	394 (48.34%)
7. Overuse of antibiotics can lead to antibiotic resistance	512 (62.82%)	37 (4.54%)	266 (32.64%)
8. Antibiotic resistance does not develop as long as it is used for a short time	340 (41.72%)	195 (23.93%)	280 (34.36%)

In terms of constructs based on the HBM (Table 4), the respondents indicated that they perceived a moderately high level of threat from AR (mean = 2.46, SD = 0.64) and a nearly moderate level of threat from URTIs (mean = 2.13, SD = 1.04). The difference between the two kinds of threat levels was statistically significant (p < 0.001). Regarding the perceived value of antibiotic use behaviors, the responses showed that the participants perceived both benefits (mean = 2.57, SD = 0.68) and potential harms (mean = 2.16, SD = 0.83) of antibiotic use, and the potential benefits were significantly higher than the potential harms (p < 0.001). In addition, the respondents had easy access to antibiotics (mean = 2.38, SD = 0.80) but had a moderate level of self-efficacy in using antibiotics (self-efficacy score = 1.97, SD = 0.75). They also perceived social influence from others (family, friends and pharmacies), and people from their surroundings showed prevalent use of antibiotics (mean = 2.40, SD = 0.65).

**Table 4:** Responses to various dimensions of antibiotic use and irrational use behaviors.

Measurement	Number of items	Score (mean ± SD)	Cronbach’s alpha
Knowledge about antibiotics	8	2.33 ± 1.71	0.738
Irrational antibiotic use behaviors	9	2.95 ± 2.12	0.703
Perceived threat of AR	5	2.46 ± 0.64	0.792
Perceived threat of URTIs	3	2.13 ± 1.04	0.893
Perceived benefits of antibiotics	4	2.57 ± 0.68	0.787
Perceived harm of antibiotics	3	2.16 ± 0.83	0.658
Antibiotic use self-efficacy	5	1.97 ± 0.75	0.780
Antibiotic availability	3	2.38 ± 0.80	0.654
Social influence	2	2.40 ± 0.65	0.727

### SEM of antibiotic use behaviors based on HBM

**Fig. 2:**
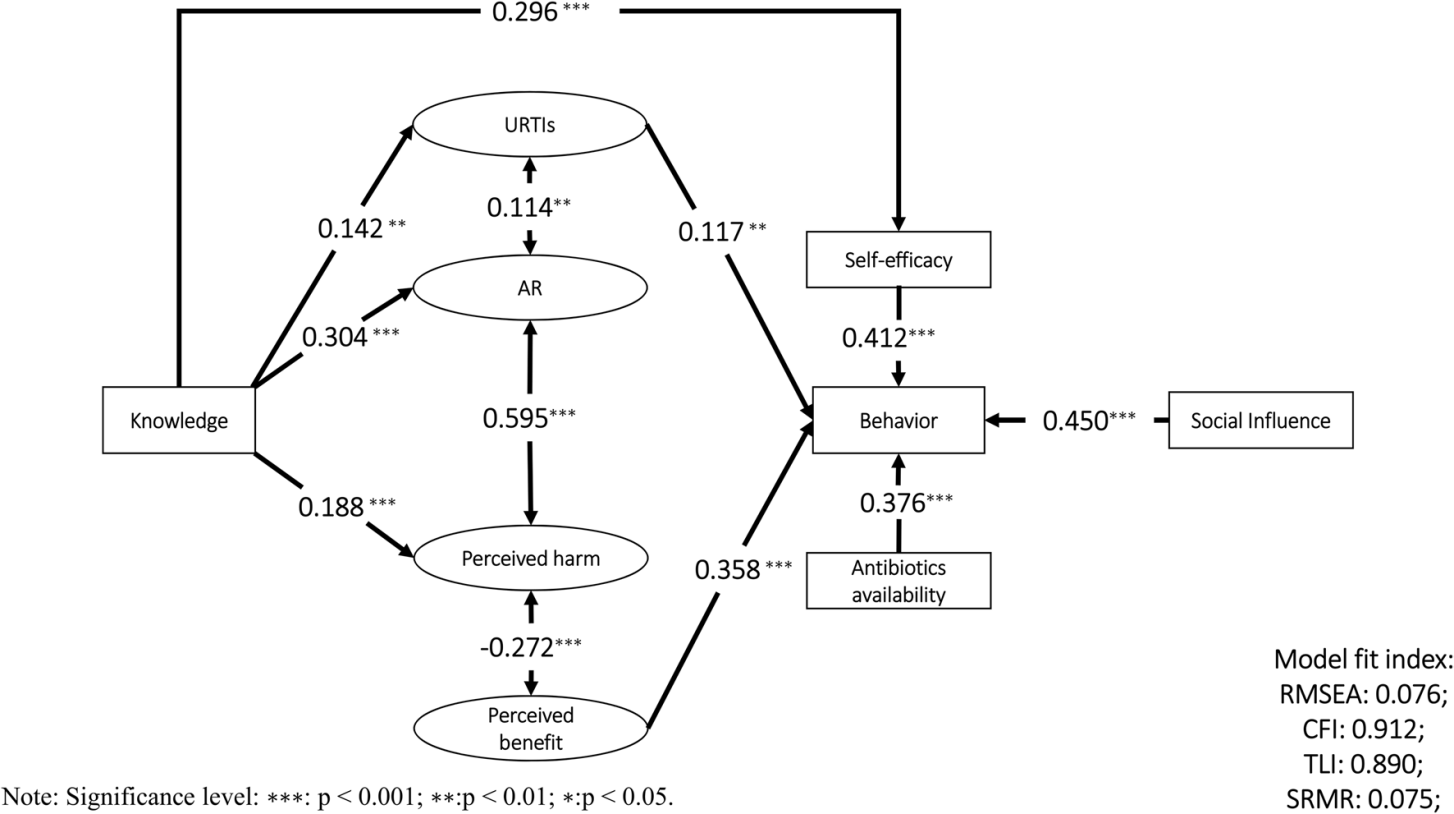
The public’s antibiotic use behaviors using a structural equation model based on HBM. *Only significant pathways (p < 0.05) are reported with standardized path coefficients.

SEM confirmed that the survey data fit the HBM model well (Fig. 2), with most fitness indices meeting the criteria (RMSEA = 0.076, 90% CI 0.073–0.079, CFI = 0.912, TLI = 0.890).

According to the results of SEM, a higher level of perceived threat of URTIs (β = 0.117, p < 0.005) and a higher level of perceived benefits of antibiotics (β = 0.358, p < 0.001) were associated with a higher level of irrational antibiotic use behaviors. Moreover, higher self-efficacy (β = 0.412, p < 0.001), higher antibiotic availability (β = 0.376, p < 0.001), and a higher level of social influence (β = 0.450, p < 0.001) were associated with more irrational antibiotic use behavior.

A higher level of knowledge was found to be linked with a higher perceived threat of URTIs (β = 0.142, p < 0.005) and AR (β = 0.304, p < 0.001) and higher perceived potential harm of antibiotics (β = 0.188, p < 0.001) but not more perceived benefits of antibiotics. However, the perceived harm of antibiotics and the perceived threat of AR were not associated with irrational antibiotic use behaviors. In addition, higher knowledge scores were associated with higher self-efficacy (β = 0.296, p < 0.001), which was associated with more irrational antibiotic use behaviors (β = 0.412, p < 0.001).

For each irrational antibiotic use behavior, SEM was conducted to confirm the robustness of the results. The results from SEM of nine different irrational antibiotic use behaviors were generally consistent, although a higher level of perceived potential harm of antibiotics was associated with respondents’ greater likelihood of adopting early cessation of antibiotic use when they felt better (β = 0.465, p < 0.001). In addition, irrational antibiotic use behaviors were affected by the level of education; the higher the respondent’s level of education was, the lower his or her level of irrational antibiotic use behaviors (β = -0.232, p < 0.05).

## Discussion

### Main findings

Based on the HBM, this study aimed to explore the reasons for the public’s irrational use of antibiotics for URTIs in western China using SEM. We found that the public’s antibiotic use is still suboptimal but has improved compared with the results from previous studies^19^. However, the public’s understanding of antibiotics and their rational use is still poor, and misconceptions of the indications for antibiotic use and causes of AR remain prevalent. People had a relatively positive assessment of antibiotic use, perceived a higher threat of AR and reported easy access to nonprescription antibiotics. The results from SEM confirmed that the public’s irrational use of antibiotics was associated with the perceived benefits of antibiotics and greater availability of these medicines, a perceived higher threat of URTIs, more social influence from relatives and higher self-efficacy of the rational use of antibiotics. In addition, greater knowledge of antibiotics may indirectly increase people’s irrational antibiotic use by increasing their self-efficacy and the perceived threat of URTIs.

### Strengths and limitations

In this study, we adopted the HBM as the theoretical basis, which enabled us to comprehensively explore potential factors that influence the public’s irrational use of antibiotics. The use of HBM helped us to partly overcome the main drawback of existing studies based on KAP^23^. Additionally, the use of SEM helped us simultaneously estimate complex relationships among various dependent and independent variables and account for measurement error to produce a more precise estimation. Finally, compared with studies using proxy indicators^24^, the current study developed a reliable and validated questionnaire to directly measure the key constructs derived from the HBM, which can reduce the potential bias due to measurement.

There are several limitations of this study. (1) The study was conducted in one province (Chongqing) of China. Attempts to generalize the findings of this study should be conducted with caution. (2) The HBM model concentrates on individual behaviors relevant to health and has some shortcomings. For example, this model could not consider the influence of contextual factors, such as cultural differences and urban–rural disparities, on people’s irrational antibiotic use behavior^47,48^. (3) Although we limited potential participants to people who had experienced URTIs in the past 6 months, self-reported data carry inherent limitations. First, recall bias may have occurred as participants struggled to accurately remember details of past antibiotic use (e.g., dosage duration). Second, social desirability bias likely led to underreporting of non-prescription antibiotic purchases, given the stigma associated with self-medication in Chinese healthcare contexts. These dual biases could result in systematic underestimation of actual misuse rates, particularly for sensitive behaviors like antibiotic sharing or prophylactic use.

### Comparison with existing studies

Based on the results of the current study, it seems that the level of irrational antibiotic use behaviors in the population of Chongqing is lower than that reported in previous studies from Central Asia, Europe, and East Asia, where the prevalence of public purchases of antibiotics without a prescription ranged from 50 to 73% and the prevalence of demand for antibiotic use ranged from 17 to 78%^27,49^. One plausible reason for the much lower level of irrational antibiotic use observed in this study may be the efforts of the Chinese government to reduce the overuse and misuse of antibiotics in recent years. Both this study and previous research conducted in Western China showed a significant decrease in the prevalence of irrational antibiotic use among the population. Since 2016, China has implemented the National Action Plan on Antimicrobial Resistance. In addition to providing health education to the public^50^, China’s AMR national action plan focuses more on regulating non-prescription drug sales and ensuring rational prescribing of doctors. In recent years, it was shown that a gradual decrease in the antibiotic prescribing rate of doctors, and regulatory measures have become increasingly stringent, reducing the likelihood of the public to obtain non-prescription antibiotics. Furthermore, the zero-mark-up policy, aiming to eradicate the potential incentive for hospitals and doctors^51^, may also play a role in reducing doctors unnecessary prescribing of antibiotics and, in turn, changing the public’s attitudes and behaviors of antibiotic use. Existing research suggests that comprehensive interventions can yield better results, and these integrated strategies may effectively reduce the public’s irrational use behavior^52^.

However, the public’s belief regarding the effectiveness of antibiotics for URTIs is still prevalent. Our study found that the perceived benefits of antibiotic use outweighed the perceived potential risks among the general public. Several previous studies have yielded conclusions similar to ours^16,21,53^. In contrast, a study^54^ conducted in the United States revealed that while most individuals acknowledged the benefits of antibiotics, their emotional response toward the use of antibiotics was mostly negative. People in China generally believe that antibiotics can prevent and treat URTIs and consider their benefits to be greater than their side effects^16^. This further underscores the widespread acceptance of antibiotic use for URTIs in China. Previous studies have demonstrated that the perceived benefits of antibiotics can lead people to use them irrationally^16,23^. Our study did not yield a significant conclusion, potentially due to the introduction of more influential factors such as self-efficacy. Overall, it is important for the public to shift their perception of antibiotics away from their perceived benefits and toward a greater awareness of their potential side effects and the risks of AR.

On the other hand, this study indicated that the public’s beliefs regarding diseases are strongly associated with irrational antibiotic use behaviors. Previous studies have shown that individual perception of risk can prevent the occurrence of misbehavior or reduce its severity, and subjects who perceive a high level of threat are less likely to self-medicate^24,55^. However, this is not the situation for antibiotic use. The current study showed that the perceived threat of AR did not translate into more appropriate antibiotic use behaviors. In contrast, the perceived threat of URTIs promoted inappropriate antibiotic use behaviors. This result was consistent with the findings of studies from China and Canada^22,23,48^. Individuals are more inclined to self-administer antibiotics to alleviate immediate symptoms of URTIs when they perceive their symptoms to be more severe. This behavior is especially apparent among parents, who may do so for their children^7^. Furthermore, health decision-making is a complex process, and individuals need to weigh the pros and cons^56^. The current study showed that individuals perceived a higher level of threat from AR than from URTIs. However, this perceived AR threat did not translate into more appropriate behaviors. A previous systematic review suggested that people commonly showed optimism about the future^19^. Instead of worrying about the future, priority is given to the recovery of current conditions. A study from France also confirmed this point^57^. Individuals often view AR as a distal threat affecting ‘others’ in an ‘uncertain future’, while relying on techno-optimism—the belief that scientific innovations (e.g., new antibiotics) will ultimately resolve the problem, thereby diminishing the sense of urgency. Therefore, it is necessary to emphasize the serious consequences of AR and associate it with people’s daily lives while emphasizing that AR is not a distant future threat.

The public’s antibiotic-related knowledge remains highly limited. Previous studies from China, the United States, and Spain have shown that the average correct response rate for knowledge of antibiotics among the general public ranges from 52 to 79%^27,58^. In our study, the knowledge level of the Chinese population was found to be even worse. While it is commonly believed that improving public knowledge can help promote rational antibiotic use, our study found contradictory results. Higher levels of knowledge did not directly influence public antibiotic use behavior. However, the increase in antibiotic knowledge may translate to increased individual self-efficacy for antibiotic use and the perceived threat of URTIs, thereby promoting irrational antibiotic use. These findings are consistent with previous studies conducted in the United Kingdom, Lithuania, and China, in which improved knowledge was associated with worse antibiotic use behavior^18,23,59^. This study explains why people with higher antibiotic knowledge levels may overestimate their ability to use antibiotics or overestimate the threat of URTIs, leading to self-diagnosis and unnecessary antibiotic use. These results may also explain why some educational interventions targeting public knowledge of antibiotics have had limited effectiveness.

Finally, the high accessibility of antibiotics in our study setting further exacerbated people’s irrational use of antibiotics. This study confirmed that the public can easily obtain antibiotics from pharmacies or relatives and friends. People are not required to produce corresponding prescriptions when purchasing from pharmacies, which is consistent with the results of a previous systematic review^23,27,60^. Furthermore, the social environment is very important. Recommendations from friends, family and pharmacies directly promote irrational use of antibiotics^61,62^. Some previous studies have suggested that culture plays a role in the public’s antibiotic use behaviors^47,63^ and that people are more inclined to believe that it is acceptable to use antibiotics when their relatives demonstrate frequent use of antibiotics.

### Policy implications

Based on the current study, a multifaceted intervention is proposed to help reduce the public’s irrational use of antibiotics. First, the education of the public on the rational use of antibiotics should be more targeted and accurate. Although many countries have issued national action plans to curb bacterial resistance, educating the general population is a core measure in these policies. Existing interventions often teach the indications for antibiotic use (bacteria vs. viral) and the consequences of the irrational use of antibiotics via printed materials or mass media^52^. According to the current study, general information regarding antibiotic use seemed to induce irrational rather than rational use of antibiotics. Interventions should be more accurate and should target the public’s perceived threat of URTIs and the prevalent misconceptions of the benefits of antibiotic use while reducing the public’s self-efficacy of antibiotic use. For example, the educational campaign “common cold needs common sense” in Australia, which reduced the public’s perceived threat of URTIs, also demonstrated a significant reduction in the public’s self-medication with antibiotics after several years of implementation.

Moreover, it is important to restrict the high availability of unnecessary antibiotics. Although most countries have enacted policies to require prescriptions for antibiotic dispensing, these policies are often poorly implemented worldwide, especially in developing countries. Studies have shown that pharmacies have limited incentives to reject consumers’ antibiotic demands, and there are different methods for pharmacies to avoid potential supervision. Regulatory (governmental inspections), managerial (involvement of pharmacists in designing interventions and retention of prescriptions) and educational (media campaigns and education of pharmacists) interventions can help prevent nonprescription sales of antibiotics in pharmacies^64^. However, it is also important to improve the rational use of health care providers, who are an important source of antibiotic storage and self-medication. Reviews have shown that education, auditing and feedback, and social norms feedback can effectively reduce unnecessary prescriptions of antibiotics, which can also help reduce the public’s irrational use of antibiotics. In addition, implementing a prescription tracking system as part of broader IT-enabled antimicrobial stewardship strategies—such as computerized provider order entry (CPOE) and clinical decision support systems (CDSS)—can enhance real-time monitoring of antibiotic dispensing, reduce nonprescription sales by enforcing regulatory compliance, and provide audit feedback to prescribers^65^. When combined with behavioral nudges (e.g., automated alerts to pharmacists and prescribers about noncompliant prescriptions), such systems may further bridge the gap between policy enforcement and individual decision-making, complementing traditional education-based interventions^66^.

## Conclusions

The public’s irrational use of antibiotics for URTIs is prevalent. People showed poor knowledge, relatively positive assessments of antibiotic use and moderate self-efficacy for antibiotic use. They also perceived a higher threat of URTIs and AR and had easy access to antibiotics. Irrational antibiotic use was associated with people who perceived a higher threat of URTIs, had a more positive assessment of antibiotic use, had higher antibiotic availability, showed higher self-efficacy for antibiotic use and perceived higher social influence from relatives. In addition, a higher level of knowledge of antibiotic use increased rather than decreased the irrational use of antibiotics by increasing the public’s self-efficacy of antibiotic use and perceived threat of URTIs. A multifaceted intervention focusing on accurate education of the public, regulation enforcement of pharmacies and the improvement of rational prescriptions by physicians is required to help reduce the public’s irrational antibiotic use.

## Supplementary Information

Supplementary Information 1.

Supplementary Information 2.

Supplementary Information 3.

## Data Availability

The data presented in this study can be made available upon reasonable request and with permission of surveyed local institutions and governments. Requests to access the datasets should be directed to the corresponding author (liu_chenxi@hust.edu.cn). The dataset is not publicly available as it forms part of ongoing research and analysis. The data that support the findings of this study are available on request from the corresponding author, upon reasonable request.

## Declarations

### Accordance

This study followed the Strengthening the Reporting of Observational Studies in Epidemiology (STROBE) statement.
