# Supplementary material for: The public’s irrational use of antibiotics for upper respiratory tract infections: a cross-sectional study based on the health belief model
Source: Sci Rep. 2025 May 17;15:17220. doi: 10.1038/s41598-025-01767-9 (PMC12085633; doi:10.1038/s41598-025-01767-9)
Supplement: Supplementary file 2 — Supplementary Information 2. [file 41598_2025_1767_MOESM2_ESM.docx]

**Survey of the public’s knowledge, perception, and behavior regarding antibiotics.**

**Dear sir/madam：**

Greeting!

We are graduate students from XXXXXXXXXX. In order to improve the level of reasonable treatment of common cold in the public, we are conducting a study of the common cold treatment model. The survey data will only be used for academic research, and we will keep your information strictly confidential. Please read the following instructions carefully before answering. There is no correct answer to this questionnaire, please answer according to your actual situation. This questionnaire takes about 10-15 minutes. Thank you for your participation!

XXXXXX

XXXX

**Filling explanation**

Antibiotics are a general term for a class of drugs. Common antibiotics are: "penicillin class", such as amoxicillin; "Cephalosporins" such as ceftazidime; "Floxacin", such as norfloxacin, levofloxacin; "Mycin" such as penicillin, azithromycin; "Cyclins," such as tetracycline. These are all antibiotics.

Please recall a time during the past year when you had symptoms of a cold (e.g., cough, runny nose, sore throat, low-grade fever, etc.). Answer the following questions:

**Part 1: Antibiotic use behaviors**

Please note that this part of the questions **do not have the correct answer**, please fill in them according to your personal situation / attitude.

| In the past year: | No/Not aware | Yes |
| --- | --- | --- |
| 1. When a cold is not serious, use antibiotics to prevent the disease from getting worse | □ | □ |
| 2. Take some antibiotics before seeing a doctor | □ | □ |
| 3. Go straight to the drugstore and buy some antibiotics before seeing a doctor | □ | □ |
| 4. Offer to ask your doctor for antibiotics | □ | □ |
| 5. The dose of antibiotics was increased by yourself because of poor effect | □ | □ |
| 6. The dose of antibiotics was reduced by yourself because of concerns about side effects | □ | □ |
| 7. Stop taking antibiotics when you feel better | □ | □ |
| 8. Antibiotics were always available at home | □ | □ |
| 9. Shared (received/given) antibiotics with family, friends, and colleagues | □ | □ |

**Part 2: Knowledge of antibiotic use**

| Knowledge Questions | True | False | No idea |
| --- | --- | --- | --- |
| 10. Antibiotics can effectively treat most colds. | □ | □ | □ |
| 11. Antibiotics are anti-inflammatory drugs. | □ | □ | □ |
| 12. Antibiotics are effective in treating viral colds. | □ | □ | □ |
| 13. Antibiotics are effective in treating bacterial colds. | □ | □ | □ |
| 14. The human body becomes resistant to antibiotics. | □ | □ | □ |
| 15. Bacteria can become resistant to antibiotics. | □ | □ | □ |
| 16. Overuse of antibiotics can lead to antibiotic resistance. | □ | □ | □ |
| 17. Antibiotic resistance does not develop as long as it is used for a short time. | □ | □ | □ |

**Part 3: Antibiotic use decisions**

| To what extent you agree with the following statements | Very agree | Agree | Neutral | Disagree | Very Disagree |
| --- | --- | --- | --- | --- | --- |
| 18. Antibiotics can relieve the symptoms of a cold | □ | □ | □ | □ | □ |
| 19. Antibiotics will help me get over my cold faster | □ | □ | □ | □ | □ |
| 20. Antibiotics can reduce the incidence of complications of cold | □ | □ | □ | □ | □ |
| 21. Antibiotics can prevent a cold from getting worse | □ | □ | □ | □ | □ |
| 22. The side effects of antibiotics are minimal |  |  |  |  |  |
| 23. I'm worried about the side effects of antibiotics | □ | □ | □ | □ | □ |
| 24. I am concerned that taking antibiotics regularly will reduce its effectiveness | □ | □ | □ | □ | □ |
| 25. Antibiotic resistance (superbugs) is a serious problem in our country | □ | □ | □ | □ | □ |
| 26. Antibiotic resistance threatens the health of itself and its families | □ | □ | □ | □ | □ |
| 27. I'm worried that superbugs could harm me and my family | □ | □ | □ | □ | □ |
| 28. Reducing individual abuse of antibiotics plays an important role in curbing antibiotic resistance | □ | □ | □ | □ | □ |
| 29. I can help reduce the overuse of antibiotics | □ | □ | □ | □ | □ |
| 30. Every time I catch a cold, I worry about my health | □ | □ | □ | □ | □ |
| 31. Every time I catch a cold, I fear it might develop into something more serious | □ | □ | □ | □ | □ |
| 32. I often worry that a cold might make me seriously ill | □ | □ | □ | □ | □ |
| 33. I think I know enough about the appropriate use of antibiotics | □ | □ | □ | □ | □ |
| 34. I think I'm capable of taking antibiotics and dealing with milder symptoms | □ | □ | □ | □ | □ |
| 35. I usually have confidence in self-diagnosis and treatment for colds | □ | □ | □ | □ | □ |
| 36. I usually know when I need to use antibiotics | □ | □ | □ | □ | □ |
| 37. I usually know if I need antibiotics for my cold before I see a doctor | □ | □ | □ | □ | □ |
| 38. I can easily get antibiotics from the drugstore | □ | □ | □ | □ | □ |
| 39. I have never been asked to show a doctor's prescription when I go to the pharmacy to buy antibiotics | □ | □ | □ | □ | □ |
| 40. I can easily get antibiotics from family, friends, and family stocks | □ | □ | □ | □ | □ |
| 41. My friends and family recommended antibiotics to treat my cold | □ | □ | □ | □ | □ |
| 42. The drugstore recommended me to buy antibiotics for my cold | □ | □ | □ | □ | □ |

**Part 4: Personal characteristics**

| 43. Age | years |
| --- | --- |
| 44. Gender | □Male □Female |
| 45. Education | □Primary school or below □Junior high school  □High school or technical school □Junior college or bachelor  □Master degree candidate □Doctoral candidate |
| 46. Occupation | □Farmer □Worker □Student □Medical professional  □Teacher □Enterprise and public institution  □Self-employment venture □Retirement  □Unemployed □Else |
| 47. Personal or family medical background | □With medical background □Without medical background |
| 48. Insurance | □Insurance for urban workers □Insurance for urban and rural residents  □New Rural Cooperative Medical system □Else |
| 49. Family annual income (￥) | □<20,000 □20,000–40,000 □40,000–60,000 □60,000–80,000 □80,000–100,000 □100,000–120,000 □120,000–140,000  □140,000–160,000 □160,000–180,000 □180,000–200,000  □200,000–220,000 □220,000–240,000 □240,000–260,000  □260,000–280,000 □280,000–300,000 □>300,000 |
| 50. Chronic disease in person or family | □Yes □No/I don’t know |
| 51. Subjective health status | □Excellent □Good □Average □Fair □Poor |

Notes: Items and their measured characteristics were as following:

Items No.18 – 21: Perceived benefits;

Items No. 22 – 24: Perceived harms;

Items No. 25 – 29: The perceived threat of antibiotic resistance;

Items No. 30 – 32: The perceived threat of a cold;

Items No. 33 – 37: Self-efficacy in antibiotic use;

Items No. 38 – 40: Availability of antibiotics;

Items No. 41 – 42: The social influences;
