# Supplementary material for: The public’s irrational use of antibiotics for upper respiratory tract infections: a cross-sectional study based on the health belief model
Source: Sci Rep. 2025 May 17;15:17220. doi: 10.1038/s41598-025-01767-9 (PMC12085633; doi:10.1038/s41598-025-01767-9)
Supplement: Supplementary file 3 — Supplementary Information 3. [file 41598_2025_1767_MOESM3_ESM.docx]

| **Table S2 Detailed information of public’s responses to individual items of knowledge, perception, and behavior regarding antibiotics.** | | | | | |
| --- | --- | --- | --- | --- | --- |
| **Measured characteristics and corresponding items** | **Responses (N, %)** | | | | |
| **Perceived benefits:** | **Very agree** | **Agree** | **Neutral** | **Disagree** | **Very disagree** |
| 1. Antibiotics can relieve the symptoms of a cold | 91 (11.17) | 493 (60.49) | 165 (20.25) | 57 (6.99) | 9 (1.10) |
| 2. Antibiotics will help me get over my cold faster | 98 (12.02) | 466 (57.18) | 169 (20.74) | 75 (9.20) | 7 (0.86) |
| 3. Antibiotics can reduce the incidence of complications of cold | 70 (8.59) | 378 (46.38) | 229 (28.10) | 125 (15.34) | 13 (1.60) |
| 4. Antibiotics can prevent a cold from getting worse | 62 (7.61) | 385 (47.24) | 200 (24.54) | 152 (18.65) | 16 (1.96) |
| **Perceived harms:** | **Very agree** | **Agree** | **Neutral** | **Disagree** | **Very disagree** |
| 5. The side effects of antibiotics are minimal | 81 (9.94) | 217 (26.63) | 272 (33.37) | 196 (24.05) | 49 (6.01) |
| 6. I am worried about the side effects of antibiotics | 90 (11.04) | 280 (34.36) | 187 (22.94) | 189 (23.19) | 69 (8.47) |
| 7. I am concerned that taking antibiotics regularly will reduce its effectiveness | 83 (10.18) | 366 (44.91) | 192 (23.56) | 148 (18.16) | 26 (3.19) |
| **The perceived threat of antibiotic resistance:** | **Very agree** | **Agree** | **Neutral** | **Disagree** | **Very disagree** |
| 8. Antibiotic resistance (superbugs) is a serious problem in our country | 72 (8.83) | 258 (31.66) | 383 (46.99) | 94 (11.53) | 8 (0.98) |
| 9. Antibiotic resistance threatens the health of itself and its families | 66 (8.10) | 295 (36.20) | 283 (34.72) | 152 (18.65) | 19 (2.33) |
| 10. I'm worried that superbugs could harm me and my family | 102 (12.52) | 335 (41.10) | 262 (32.15) | 96 (11.78) | 20 (2.45) |
| 11. Reducing individual abuse of antibiotics plays an important role in curbing antibiotic resistance | 107 (13.13) | 377 (46.26) | 269 (33.01) | 53 (6.50) | 9 (1.10) |
| 12. I can help reduce the overuse of antibiotics | 54 (6.63) | 393 (48.22) | 302 (37.06) | 55 (6.75) | 11 (1.35) |
| **The perceived threat of a cold:** | **Very agree** | **Agree** | **Neutral** | **Disagree** | **Very disagree** |
| 13. Every time I catch a cold, I worry about my health | 81 (9.94) | 306 (37.55) | 124 (15.21) | 255 (31.29) | 49 (6.01) |
| 14. Every time I catch a cold, I fear it might develop into something more serious | 83 (10.18) | 307 (37.67) | 122 (14.97) | 260 (31.90) | 43 (5.28) |
| 15. I often worry that a cold might make me seriously ill | 81 (9.94) | 289 (35.46) | 123 (15.09) | 273 (33.50) | 49 (6.01) |
| **Self-efficacy in antibiotic use:** | **Very agree** | **Agree** | **Neutral** | **Disagree** | **Very disagree** |
| 16. I think I know enough about the appropriate use of antibiotics | 22 (2.70) | 218 (26.75) | 202 (24.79) | 287 (35.21) | 86 (10.55) |
| 17. I think I'm capable of taking antibiotics and dealing with milder symptoms | 21 (2.58) | 299 (36.69) | 175 (21.47) | 258 (31.66) | 62 (7.61) |
| 18. I usually have confidence in self-diagnosis and treatment for colds | 56 (6.87) | 367 (45.03) | 167 (20.49) | 205 (25.15) | 20 (2.45) |
| 19. I usually know when I need to use antibiotics | 25 (3.07) | 325 (39.88) | 165 (20.25) | 256 (31.41) | 44 (5.40) |
| 20. I usually know if I need antibiotics for my cold before I see a doctor | 18 (2.21) | 247 (30.31) | 165 (20.25) | 330 (40.49) | 55 (6.75) |
| **Availability of antibiotics:** | **Very agree** | **Agree** | **Neutral** | **Disagree** | **Very disagree** |
| 21. I can easily get antibiotics from the drugstore | 142 (17.42) | 369 (45.28) | 202 (24.79) | 95 (11.66) | 7 (0.86) |
| 22. I have never been asked to show a doctor's prescription when I go to the pharmacy to buy antibiotics | 99 (12.15) | 313 (38.40) | 214 (26.26) | 153 (18.77) | 36 (4.42) |
| 23. I can easily get antibiotics from family, friends, and family stocks | 71 (8.71) | 308 (37.79) | 133 (16.32) | 246 (30.18) | 57 (6.99) |
| **The social influences:** | **Very agree** | **Agree** | **Neutral** | **Disagree** | **Very disagree** |
| 24. My friends and family recommended antibiotics to treat my cold | 43 (5.28) | 303 (37.18) | 121 (14.85) | 277 (33.99) | 71 (8.71) |
| 25. The drugstore recommended me to buy antibiotics for my cold | 58 (7.12) | 338 (41.47) | 135 (16.56) | 218 (26.75) | 66 (8.10) |
